# Amygdalin as a chemoprotective agent in co-treatment with cisplatin

**DOI:** 10.3389/fphar.2022.1013692

**Published:** 2022-09-20

**Authors:** Panayiota Christodoulou, Panagiotis Boutsikos, Christiana M. Neophytou, Theodora-Christina Kyriakou, Maria-Ioanna Christodoulou, Panagiotis Papageorgis, Anastasis Stephanou, Ioannis Patrikios

**Affiliations:** ^1^ School of Medicine, European University Cyprus, Nicosia, Cyprus; ^2^ Tumor Immunology and Biomarkers Laboratory, Basic and Translational Cancer Research Center, Department of Life Sciences, European University Cyprus, Nicosia, Cyprus; ^3^ Tumor Microenvironment, Metastasis and Experimental Therapeutics Laboratory, Basic and Translational Cancer Research Center, Department of Life Sciences, European University Cyprus, Nicosia, Cyprus

**Keywords:** chemothearpy, chemoprotection, amygdalin, breast cancer, natural products

## Abstract

Amygdalin is a naturally occurring glycoside used in traditional Chinese medicine and is known to have anti-cancer properties. Even though the anti-cancer properties of amygdalin are well known, its effect on normal cells has not been thoroughly investigated. The aim of the present study was to investigate a possible chemo-protective role of amygdalin against the cytotoxic effects of chemotherapy for normal human cells. Specifically, it was tested in combination with a strong chemotherapeutic drug cisplatin. Human non-tumorigenic MCF12F epithelial cell line, human fibroblasts cells, human breast cancer MCF7 and MDA-MB-231 cells were treated with cisplatin in a dose- and time-depended manner in the absence or presence of amygdalin. When MCF12F cells and fibroblasts underwent pre-treatment with amygdalin followed by cisplatin treatment (24 h amygdalin + 24 h cisplatin), the cell viability was increased (22%, *p* < 0.001) as indicated using MTT assay. As attested by flow cytometry, combination treatment was associated with decreased the percentage of late apoptotic cells compared with monotherapy (fold-change of decrease = 1.6 and 4.5 for 15 and 20 μΜ, respectively). Also, the proteins expression of PUMA, p53, phospho-p53 and Bax decreased, when a combination treatment was used vs. cisplatin alone, while the proapoptotic proteins Bcl-2 and Bcl-xL exhibited an increased tendency in the presence of amygdalin. Moreover, the levels of pro-apoptotic genes *PUMA*, *p53*, and *BAX* mRNA were significantly downregulated (∼83%, ∼66%, and ∼44%, respectively) vs. cisplatin alone, while the mRNA levels of anti-apoptotic genes *BCl-2* and *Bcl-XL* were upregulated (∼44.5% and ∼51%, respectively), vs. cisplatin alone after 24 h of combination treatment. The study on the Combination index (CI) assay indicated that amygdalin could be possibly considered as an antagonist to cisplatin (2.2 and 2.3) for MCF12F and fibroblast cells, respectively. In contrast, for the breast cancer MCF7 and MDA-MB-231 cells, amygdalin and cisplatin indicated a synergistic effect (0.8 and 0.65), respectively. Our present findings suggest that amygdalin has chemo-modulatory effect when used in co-treatment with cisplatin and is able to protect normal breast cells as well as the fibroblasts during chemotherapy treatment, indicating a strong selective chemoprotective ability and may contribute to a better quality of life for cancer patients.

## Introduction

Cancer is the second leading cause of death worldwide, led by cardiovascular diseases (Nagai and Kim, 2017). Breast cancer (BC) is the most frequent cancer among women worldwide with a major public health concern [World Health Organization (WHO), 2021]. Chemotherapy is the most effective and frequently used treatment for the majority of malignancies (Huang et al., 2017). Despite many advances over the past decade in adjuvant therapies of BC, multiple issues remain unresolved including adverse side effects caused by chemotherapeutic drugs such as nausea, hair loss, vomiting, fatigue, and in severe cases even death (Waris and Ahsan, 2006; Ye et al., 2017). A total of 132 chemotherapeutic drugs are approved by the US Food and Drug Administration, of which 56 drugs have been reported to produce oxidative stress (Chen et al., 2018). Many classes of chemotherapeutic drugs such taxanes, and platinum derivatives can induce oxidative stress (Cauli, 2021).

Cis-diammininedichloroplatinum (II) (cisplatin) is an inorganic compound, an “alkylating agent,” used as a major treatment drug able to decrease cancer cell growth, against different human cancers including breast, testicular, ovarian, and lung (Dasari and Tchounwou, 2014). Cisplatin forms intra and inter-strand adducts with DNA, and thus it is a potent inducer of cell cycle arrest leading to apoptosis for most cancer cell types. Cisplatin crosslinking interactions with DNA promote inhibition replication, transcription and other nuclear functions that can arrest cancer cell proliferation and tumor growth (Zwelling et al., 1979; Dasari and Tchounwou, 2014). The efficacy of cisplatin depends on the ability of the cells to either repair DNA damage or proceed to death (Mirmalek et al., 2016). Therefore, the signaling pathways that regulate apoptosis have a key role on how the cells will respond to cisplatin (Eastman, 1990).

Amygdalin (D-mandelonitrile-β-Gentiobioside) is a cyanogenic diglucoside which is naturally found in the pits of numerous fruits and plants of the *Rosaceae* family such us *Prunus armeniaca* (apricot) and *Prunus persica* (peach). Mounting evidence has supported that amygdalin (that is also well known as “laetrile”) may as act as an anti-cancer agent, inducing cell cycle arrest and apoptosis (Guo et al., 2013; Makarevic et al., 2014; Lee and Moon, 2016; Saleem et al., 2018). Amygdalin exhibit synergistic effect when combined with other compounds such as hydrocyanic acid (an antitumor substance) and benzaldehyde (an analgesic compound); inducing cell death in cancer cells (Song and Xu, 2014). Furthermore, *in vitro,* amygdalin has been reported associated with anti-cancer activity vs. breast cancer cells mainly through oxidative stress; promoting differential inhibition in MCF7 and T470 cell proliferation (Abboud et al., 2019).

Amygdalin can be broken down by an enzyme known as *β*-Glucosidase releasing hydrogen cyanide, benzaldehyde, and glucose. Benzaldehyde is a pain killer that can be converted to benzoic acid by oxygen, within normal/healthy tissues. Hydrogen cyanide can induce cyanide toxicity and therefore kill the cancer cells (Blaheta et al., 2016). On the other hand, a different enzyme, the rhodanese, that is present only in normal tissues and not in cancerous, seems to have the ability to detoxify cyanide and therefore protect the normal tissues (Newmark et al., 1981). These two aforementioned enzymes can probably contribute to the amygdalin’s selective toxicity controlling the growth and metastasis of cancer cells.

Now we are aiming to investigate the effect of amygdalin, cisplatin and their combination treatment on cell viability using both normal and cancer cell lines and to evaluate the potential chemoprotective role of amygdalin.

## Materials and methods

### Cell culture

Amygdalin was purchased from Sigma-Aldrich (St. Louis, Missouri, United States), and dissolved in water (stock 1M). Cisplatin 1 mg/ml concentrate for solution for infusion was purchased from Accord. MCF12F, MCF7 and MDA-MB-231 cell lines were purchased from American Type Culture Collection (ATCC, Rockville, MD). Fibroblasts extracted from pancreatic tissue were a kind gift by University of Cyprus. MCF12F and fibroblast cells were cultured in Dulbecco’s modified essential media with Ham’s F-12 nutrient mix (DMEM/F12) containing 5% Chelex-treated horse serum purchased by Sigma-Aldrich, epidermal growth factor (EGF, 10 μg/500 ml), cholera toxin (50 μg/500 ml), insulin (5 mg/500 ml) and hydrocortisone (250 μg/500 ml) along with 1% antibiotics and antimycotics. MCF7 and MDA-MB-231 cells were culture in DMEM high glucose medium containing 10% Fetal Bovine Serum and 1% antibiotic purchased by Sigma-Aldrich. Cells were incubated at 37°C in a humidified chamber at 95% O_2_/5% CO_2_. Bcl-2, phospho-p53, p53, Bax, GAPDH, Caspase-9, antibodies were purchased from Cell Signalling Technology (Danvers, Massachusetts, United States). *β*-Actin and Caspase-8 were purchased from Santa Cruz Biotechnology Inc. Cell culture reagents (DMEM, FBS, HS, antibiotic/antimycotic and trypsin) were purchased from Gibco, Invitrogen (Carlsbad, California, United States).

### Combination index analysis

Combination Index (CI) assay is the simplest way to assess pharmacological drug interactions and in our case was used to quantify synergism or antagonism. Synergism is used to describe the improvement of tumor response while antagonism is used when the effect of combination is less toxic than the result of individual effects.

The results of Combination Index assay are based on the theory of Chou-Talalay (Chou, 2010). CompuSyn is a computer program for quantitation of synergism and antagonism in drug combinations and the determination of IC50 and ED50 values. When this specific assay gives CI = 1 means the substances that react have additive effect, CI < 1 means the substances that react have synergistic effect and CI > 1 means the substances that react have antagonistic effect (Table 1 and Supplementary Material 2) (Chou, 2010).

**TABLE 1:** IC50 values of different cell lines.

Cell line	Cisplatin (μΜ)	Amygdalin (mΜ)	Combination index (CI)^a^
MCF12F	23.8 ± 1.83	85.4 ± 1.78	2.2
MCF-7	21.7 ± 1.73	64.5 ± 1.86	0.8
FBS	23.9 ± 1.95	93.8 ± 1.44	2.3
MDA-MB-231	18.6 ± 1.69	69.9 ± 1.52	0.65

a For combination of 15 μM Cisplatin and 10 mΜ amygdalin.

IC50 values of breast cancer (MCF7 and MDA-MB-231) and normal (MCF12F) cell lines as well as in Fibroblasts (FBS), and the effect of cisplatin and amygdalin alone and in combination in breast cell lines at 48 h. The data are expressed as the mean (±SE) of the results from three separate experiments. Combination index >1 indicates an antagonistic effect while CI < 1 suggests that compounds are acting in a synergistic manner.

### MTT assay

For cell viability assessment, MTT [3-(4,5-dimethylthiazole-2-yl)-2,5-diphenyl tetrazolium bromide, M2128 from Sigma-Aldrich] cell proliferation assay was carried out (Gasparini et al., 2017). After trypsinization and counting with a hemocytometer, MCF12F and MCF7 cell were seeded in 96-well plate (Green and Sambrook, 2019). Once adhesion was verified (after about 18 h post-seeding), cells were incubated with 10 mM of amygdalin and after 24 h were added 15 μM of cisplatin for another 24 h. After cell treatment with 20 μl of MTT dye for 4 h and then incubated with 150 μl of DMSO (dimethyl sulfoxide, D8418 from Sigma-Aldrich) for 15 min. Absorbance at 595 nm was measured with a microplate reader (ThermoFisher Scientific).

### cDNA synthesis

The total RNA from each cell population was isolated by using of RNeasy Micro Kit (50) and the concentration as well as the purity was measured by using the absorption in *λ* = 280 and 260 nm/280 nm, respectively. The cDNA of each sample is synthesized by primeScript first strand cDNA Synthesis kit (Takara) for mixture 1 and 2. The mixture 1 was incubated in 65°C for 10 min and then in ice for 3 min before the adding of mixture 2 to final volume of 20 μl. The last step was the incubation of the mixtures in specific temperatures in RT-qPCR machine (Bio-Rad) (Neophytou et al., 2019).

### RT-qPCR

For measuring mRNA expression, real time PCR (RT-qPCR) was performed by using the KAPA SYBR FAST qPCR kit (KK4610). The following primers were used: Bax, forward: 5′-ACA​TGG​AGC​TGC​AGA​GGA​TG-3′, reverse: 5′-CCA​GTT​GAA​GTT​GCC​GTC​AG-3′; p53, forward: 5′-CCT​CAG​CAT​CTT​ATC​CGA​GTG​G-3′, reverse: 5′-TGG​ATG​GTG​GTA​CAG​TCA​GAG​C-3′; PUMA, forward: 5′-ACG​ACC​TCA​ACG​CAC​AGT​ACG​A-3′, reverse: 5′-GTA​AGG​GCA​GGA​GTC​CCA​TGA​T-3′; Bcl-2, forward: 5′-GGA​TAA​CGG​AGG​CTG​GGA​TG-3′, reverse: 5′-GGC​CAA​ACT​GAG​CAG​AGT​CT-3′; Bcl-xL, forward: 5′-AGA​GCC​TTG​GAT​CCA​GGA​GA-3′, reverse: 5′-TCA​GGA​ACC​AGC​GGT​TGA​AG-3′; GADPH, forward: 5′-GTC​TCC​TCT​GAC​TTC​AAC​AGC​G-3′, reverse: 5′-ACC​ACC​CTG​TTG​CTG​TAG​CCA​A-3′. GADPH was used as a housekeeping gene. At the end of the reaction, Ct values were recorded, and mean average of triplicates were used to calculate gene expression fold change using the 2[-Delta Delta C(T)] method (Livak and Schmittgen, 2001), as previously described (Papageorgis et al., 2010). Data from at least three independent biological replicates were used to assess the effect of treatment.

### Protein extraction

The medium was removed from the 6-well plate and 1 ml of PBS was added to wash the wells and then was aspirated. After that 150 μl RIPA were added fore lysis of the cells. The homogenized samples were transferred to Eppendorf tubes in ice for 30 min with frequent vortex. Samples were centrifugated at 10.000 rpm/10 min/4°C (Ngoka, 2008). Samples were stored at –80 until further use (Supplementary Materials 1 and 3).

### Western blotting

To determine protein levels, we performed Western blot analysis. The protein mixtures were incubated in 98°C for 5 min and then they transferred to PVDF membrane and blocked in 5% skimmed milk for 1 h in room temperature. Membranes were incubated in primary antibody overnight at 4°C and then the secondary antibody was added for 60 min in room temperature. MCF12F were treated with cisplatin and amygdalin alone or in combination for 48 h. Proteins were extracted and separated *via* 10% sodium dodecyl sulfate-polyacrylamide gel electrophoresis and probed with antibodies against Bcl-2, phospho-p53, p53, Bax, PUMA, PARP-1, and caspase-9. Bcl-2 (CST 15071, 1:1,000), PARP-1 (CST 9542, 1:1,000), Bax (CST 2772, 1:1,000), caspase -9 (CTS 9502, 1:1,000), phospho-p53 (CST 9286 (Ser15), 1:1,000), p53 (CST 9282, 1:1,000), PUMA (CST 12450, 1:1,000) were purchased for Cell Signalling Technology (Danvers, Massachusetts, United States) and Bcl-2 (sc-783, 1:1,000), GAPDH (sc-25778, 1:1,000), were purchased from Santa Cruz Biotechnology Inc. We visualised the bands of the proteins using an enhanced chemiluminescence (ECL) Western blotting substrate (Thermo Fisher Scientific) and the Chemidoc machine by Biorad. The intensity values from the densitometry analysis of Western blots were normalized against GAPDH or *β*-Actin using ImageJ analysis software. Intensity values were expressed as fold change compared to control (Ngoka, 2008).

### Apoptosis/necrosis assessment by flow cytometry

The study of apoptosis/necrosis induction was estimated by flow cytometric analysis, upon double staining with Annexin-V-FITC/propidium iodide (PI). Cells were seeded at a concentration of 1 × 10^5^ cells per well in 6-well tissue culture plates and treated with cisplatin (15 μM, 20 μM, 30 μM) and/or amygdalin (10 mM) as indicated. Cells treated only with cisplatin were used as a positive control, while untreated cells as a negative control. Cells were harvested and stained using the Annexin V/Dead Cell Apoptosis kit (Life Technologies, UK), following manufacturer’s instructions. Cell apoptosis and necrosis were analyzed using the Attune NxT flow cytometer (Thermo Fisher Scientific, UK) and the FlowJo v10 software (BD Biosciences, United States) (Steensma et al., 2003).

### Statistical analysis

All the results were presented as Mean ± Standard Error between the lowest and highest points of measurement. Unpaired *t*-tests were applied to investigate possible differences in continuous variables for two-group. *p*-values presented as two-tailed with confidence intervals of 95%. The statistical test and analysis were conducted using Prism software version 5.0 (GraphPad, San Diego, California, United States). For drug interaction the Chou-Talalay method (Combination Index) was used to evaluate the effect of combination treatment based on concentration-effect data (Chou, 2010). This method for drug combination is based on the median-effect equation that comes from the mass-action law principle that links single entity and multiple entities, and first order and higher order dynamics (Chou, 2011). For combination index equation software results CompuSyn program is used (Chou, 2010).

## Results

### Assessment of cell viability of normal and cancer breast cells following single treatment with cisplatin or amygdalin

To determine the effect of amygdalin and cisplatin treatment on normal (MCF12F) and cancer (MCF7, MDA-MB-231) breast cells as well as in fibroblasts (FBS), we used the MTT Assay to assess the cell viability in a dose-dependent manner at different time-points. Firstly, the effect of amygdalin and cisplatin was assessed separately. When the ideal concentration (cisplatin 15 μM, amygdalin 10 mM) for both treatments was specified, a combination treatment using cisplatin and amygdalin together was performed to evaluate the difference in cell number.

**FIGURE 1:**
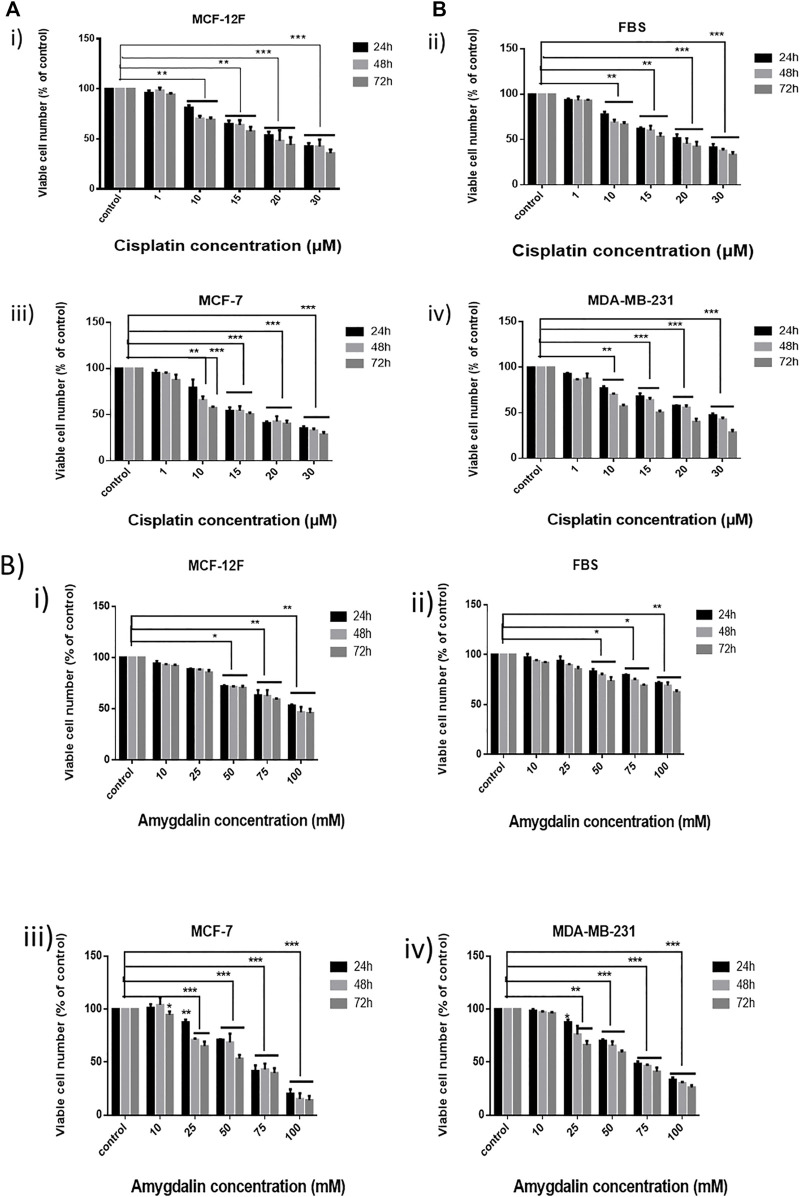
Effect of cisplatin and amygdalin in breast cell lines as well as in fibroblasts. **(A)** MTT assay was employed for the cytotoxicity evaluation (% cell viability) of increasing concentrations of cisplatin (1, 10, 15, 20, and 30 μM) in i) MCF-12F, ii) FBS, iii) MCF-7, and iv) MDA-MB-231 for 24, 48, 72 h and **(B)** the increasing concentrations of amygdalin (10, 25, 50, 75, and 100 mΜ) in i) MCF-12F, ii) FBS, iii) MCF-7, and iv) MDA-MB-231 for 24, 48, 72 h treatment. The asterisks indicate the strength of statistical significance between bars [* (0.05) < ** (0.01) < ***(0.001)].

MCF12F, MCF7, MDA-MB-231, and FBS cells were treated with 1, 10, 15, 20, and 30 μΜ of cisplatin and cell viability was assessed in 24, 48, and 72 h (Figure 1A). Cisplatin affected all cell lines by reducing the number of viable cells. Breast cancer cells were affected more by cisplatin than breast normal cells as well as in fibroblasts. However, both cell lines were significantly affected by cisplatin.

Furthermore, MCF12F, MCF7, MDA-MB-231, and FBS cells were treated with 10, 25, 50, 75, and 100 mM of amygdalin for 24, 48, and 72 h (Figure 1B). In all cell lines, the viable cell number decreased with increasing concentration of amygdalin. However, there was no significant difference using 10 mM of amygdalin treatment as the cell viability rate is >90% in all timepoints. The IC_50_ of cisplatin was determined to be 23.8 μM in MCF12F and 21.7 μM in MCF7, while the IC_50_ of amygdalin was 85.4 mM in MCF12F, and 64.5 mM in MCF7 after 24, 48, and 72 h of treatment, respectively (Table 1).

### Combination treatment shows a chemoprotective effect in breast normal cells

**FIGURE 2:**
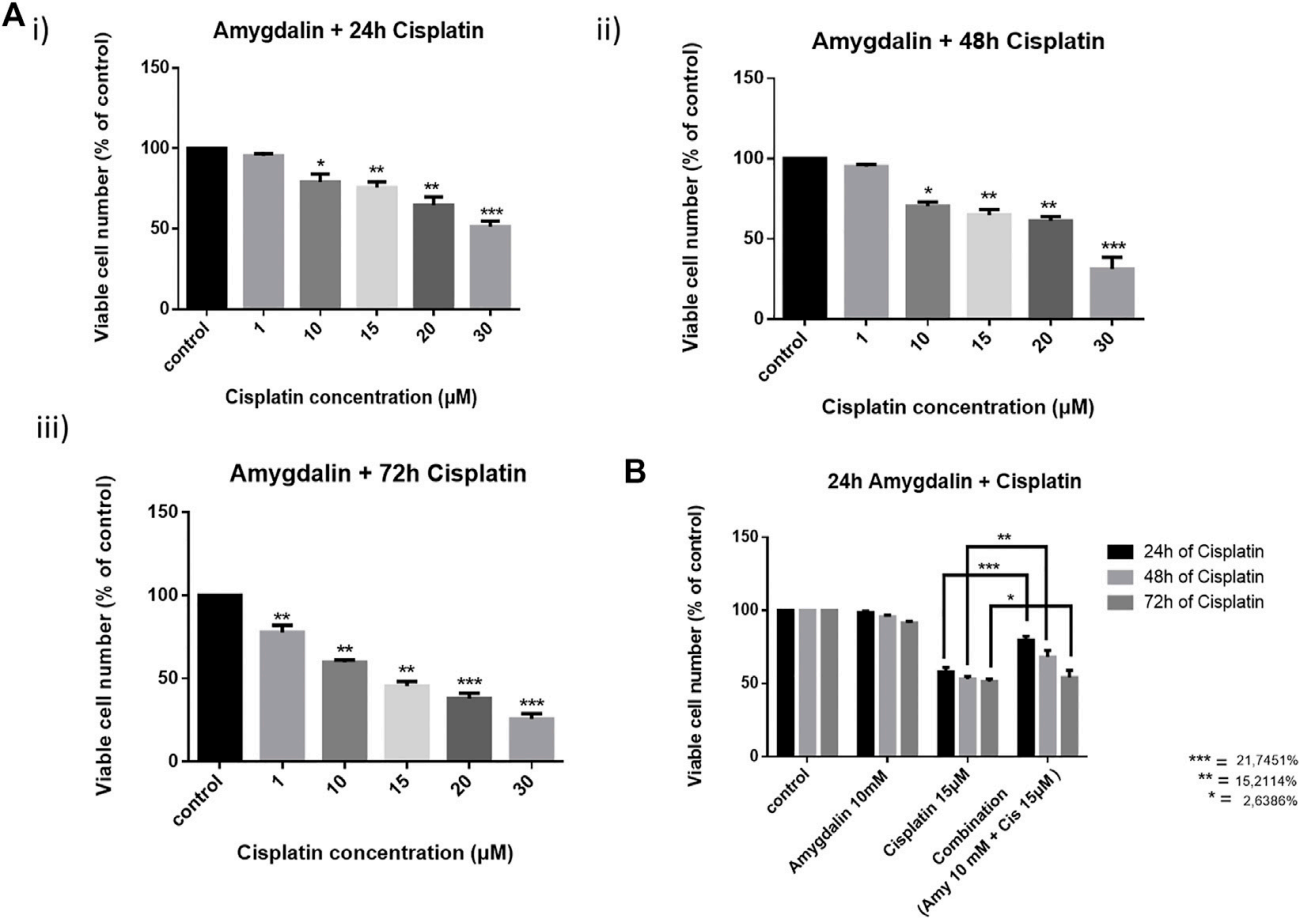
Effect of cisplatin alone and in combination with amygdalin in normal breast cells, MCF12F. Cells were pre-treated with 10 mM of amygdalin and then cisplatin (1, 10, 15, 20, and 30 μM) was added for **(A)** i) 24 h, ii) 48 h, and iii) 72 h. The MTT assay was applied to measure viability of cells under these conditions. **(B)** Amygdalin (10 mM) reduces the cytotoxicity of cisplatin (15 μM) and increases survival at all time points with the pick at 48 h of total treatment. The results represent the mean ± SEM of three different replicates and are representative of at least three different experiments [* (0.05) < ** (0.01) < ***(0.001)].

Combination treatment was applied in MCF12F cells using 10 mM of amygdalin and 1, 10, 15, 20, and 30 μM of cisplatin. The cells were first treated with amygdalin for 24 h and then cisplatin was added for 24, 48, and 72 h (Figures 2Ai–iii). We then pre-treated cells with 10 mM of amygdalin for 24 h and with the most appropriate cisplatin concentration, 15 μM for 24, 48, and 72 h. The concentration of cisplatin was based on the cell viability which was >50% when treated with the single agent. The results demonstrate that combination treatment increases the viability of the cells in all timepoints compared to cisplatin treatment alone (Figure 2B). The most significant increase in cell viability (∼22%) was observed in 48 h upon combination treatment (24 h amygdalin + 24 h cisplatin). Combination Index analysis indicating the synergism or antagonism of amygdalin with cisplatin are shown in Table 1.

### Evaluation of apoptosis mediated by combination treatment

Annexin V/PI staining was employed for the evaluation of the apoptotic effect of increasing concentrations of 15 and 20 μM cisplatin with or without 10 mΜ amygdalin for 48 h treatment.

**FIGURE 3:**
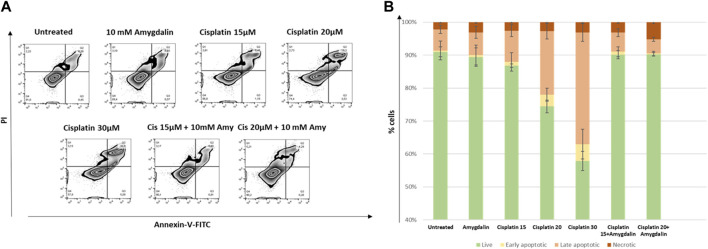
The effect of amygdalin in the apoptosis induced by cisplatin in MCF-12F cells. **(A)** Annexin V/PI staining was employed for the evaluation of the apoptotic effect (% compared to control) of 15 and 20 μM cisplatin with or without 10 mΜ amygdalin for 48 h. Zebra plot diagrams of the % of live (Q4), early (Q3), late apoptotic (Q2) and necrotic cells (Q1) in each stimulation are reported. **(B)** Stacked bar diagrams depicting the changes in the aforementioned populations. Data of three independent experiments are shown.

As depicted in Figure 3, in MCF12F cells, the % percentage of late apoptotic cells (Annexin-V/PI double positive) following 48 h-combination treatment (%mean ± SE; Cisplatin 15 μM + 10 mM amygdalin: 5.8 ± 1.2 or cisplatin 20 μM + 10 mM amygdalin: 4.3 ± 0.6) was significantly lower compared with cisplatin treatment (15 μM: 9.5 ± 1.8; fold-change of decrease = 1.6 or 20 μM: 19.3 ± 2.4; fold-change of decrease = 4.5). What is more, % of live cells upon combination treatment was increased (cisplatin 15 μM + 10 mM amygdalin: 90.1 ± 1.2 or cisplatin 20 μM + 10 mM amygdalin: 90.2 ± 0.5) compared with cisplatin only (15 μM: 86.8 ± 0.5; *p* = 0.05 or 20 μM: 74.4 ± 1.9; *p* = 0.03, respectively). The % percentages of live, early, late apoptotic and necrotic cells for all conditions of treatment are presented in Figure 3B. 30 μM concentration was used since it was found to be the highest concentration able to kill more than 50% of the treated cells. Furthermore, 30 μM was used as a positive control for the rest of the experiments.

### Amygdalin and cisplatin regulate apoptosis-related proteins

**FIGURE 4:**
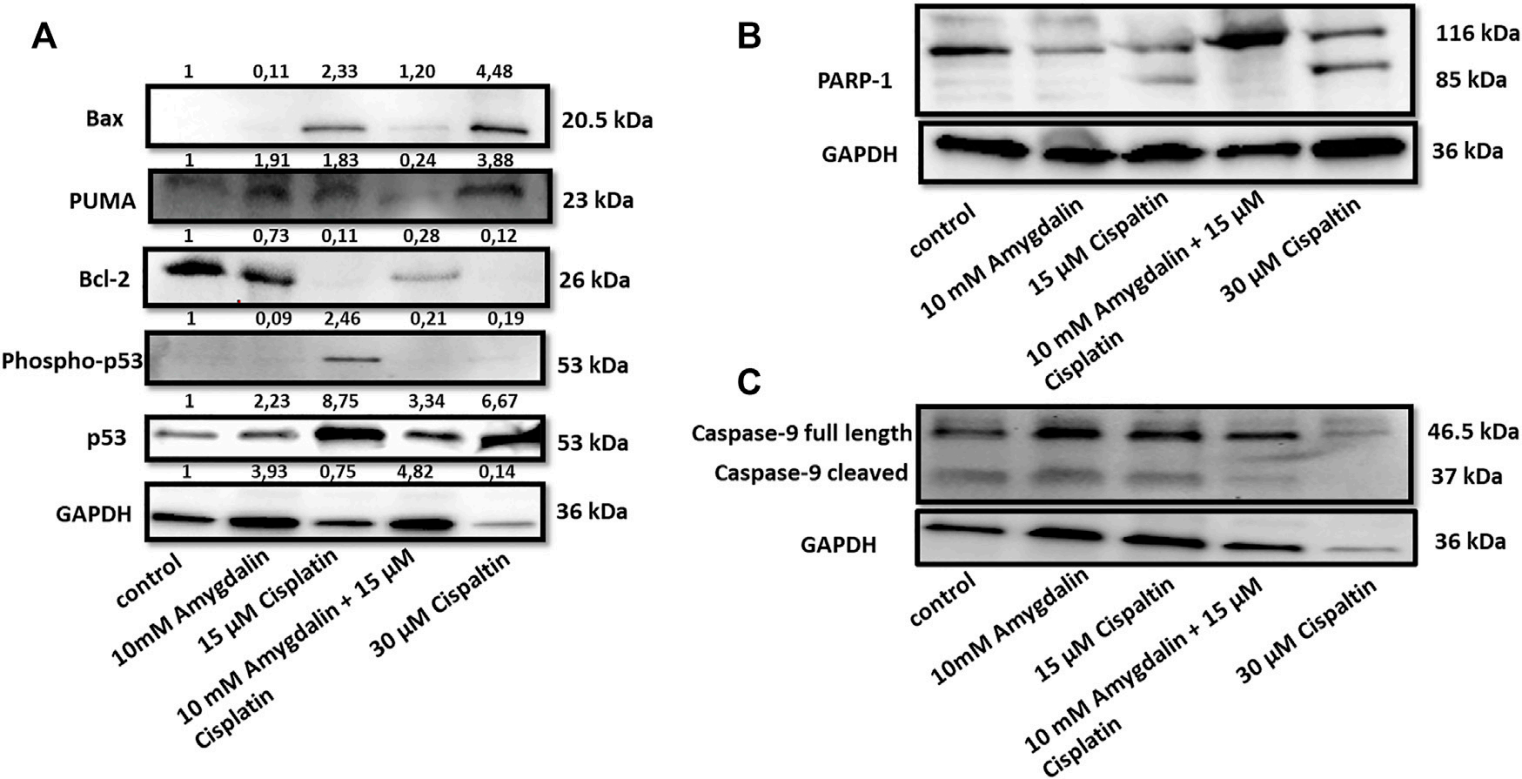
Effect of amygdalin and cisplatin on the levels and localization of apoptotic proteins in normal cells. **(A)** The combination of 10 mΜ amygdalin and 15 μM cisplatin reduced the protein levels of Bax, phosho-p53, p53, PUMA and increased the protein levels of Bcl-2 following 48 h of treatment in MCF12F. **(B)** The expression of cleaved PARP is shown under cisplatin treatment. Cisplatin alone caused cleavage of caspase-9 while **(C)** amygdalin (10 mM) reduced this effect. The intensity values from the densitometry analysis of Western blots are shown on the top of each blot and were normalized against GAPDH using ImageJ software. The results are representative of at least three independent experiments.

Combination of cisplatin and amygdalin reduced the levels of pro-apoptotic BAX, phospho-p53, p53, and PUMA, mediators of apoptotic responses (Figure 4). In contrast, the levels of anti-apoptotic BCl-2, a known inhibitor of the Mitochondrial Outer Membrane Permeabilization (MOMP) process, were increased (Figure 4A). PARP-1, a DNA-repair enzyme that is cleaved during apoptosis, did not present its cleaved form during combination treatment (Figure 4B). Cleaved caspase-9, a target of pro-apoptotic proteins released from mitochondria, was detected in cisplatin-treated cells, but not in the combination treatment group (Figure 4C). Cleaved caspase-9 is the active form that is cleaved from pro caspase-9 (full length) showing the initiation of the apoptotic pathway. These results indicate that combination treatment with amygdalin suppressed apoptosis compared to cisplatin treatment alone.

### Effect of amygdalin and cisplatin on mRNA expression of pro- and anti-apoptotic genes

**FIGURE 5:**
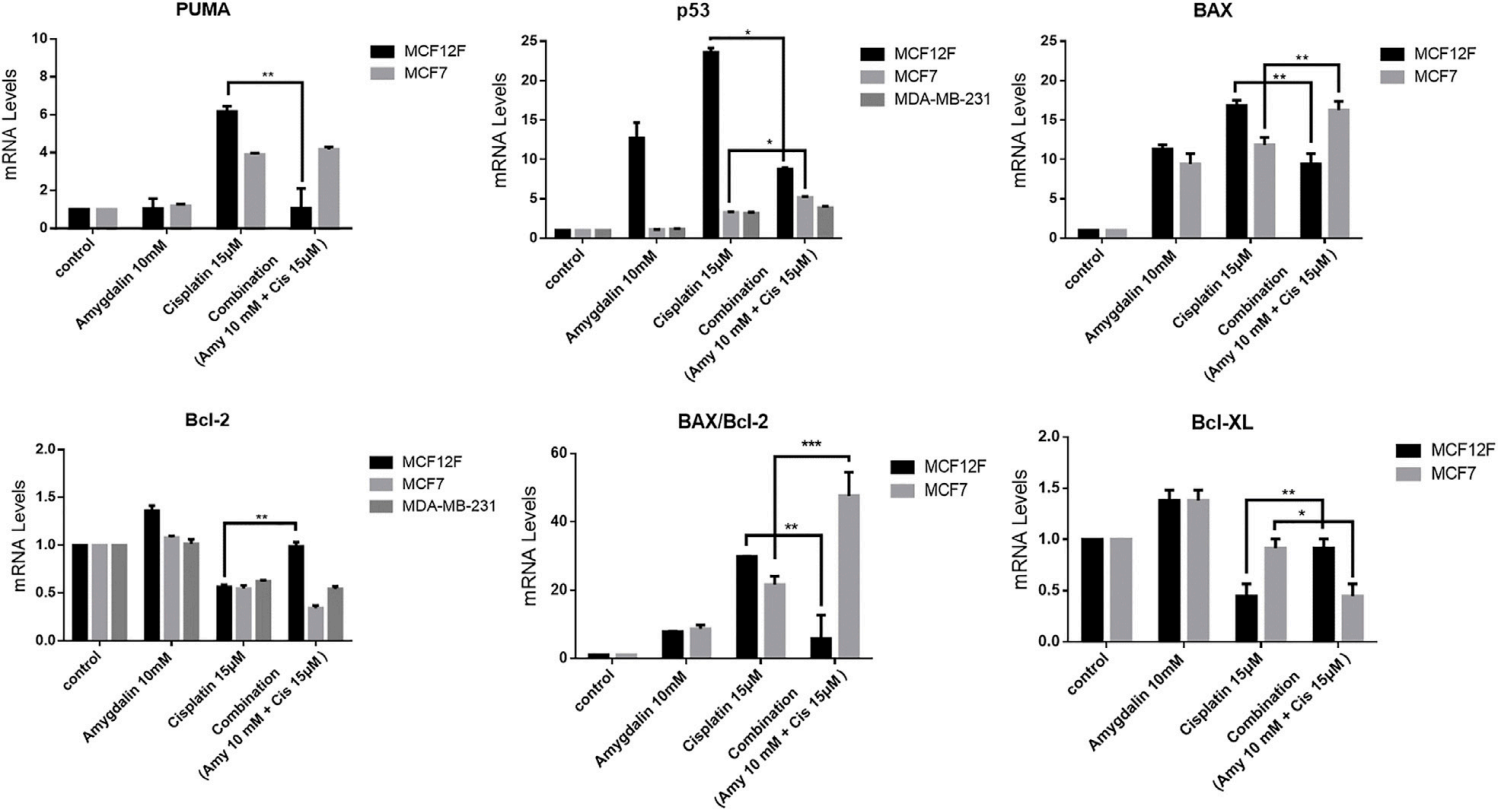
The effect of amygdalin and cisplatin on the mRNA expression of proapoptotic and antiapoptotic genes. In MCF12F the mRNA expression of pro-apoptotic genes *PUMA, p53*, and *Bax* as well as the ratio of *BAX/Bcl-2* were decreased in treatment with combination of amygdalin (10 mM) and cisplatin (15 μM), comparing with cisplatin (15 μM) treatment while the expression of mRNA of anti-apoptotic genes *Bcl-2* and *Bcl-xL* was increased in combination treatment. In MCF7 the mRNA expression of pro-apoptotic genes *PUMA, p53*, *and Bax* as well as the ratio of *BAX/Bcl-2* were increased in treatment with combination of amygdalin (10 mM) and cisplatin (15 μM), compared to cisplatin (15 μM) treatment while the expression of mRNA of anti-apoptotic genes *Bcl-2* and *Bcl-xL* was decreased in combination treatment. In MDA-MB-231 the mRNA expression of pro-apoptotic gene p*53* was increased in treatment with combination of amygdalin (10 mM) and cisplatin (15 μM), comparing with cisplatin (15 μM) treatment while the expression of mRNA of anti-apoptotic gene *Bcl-2* was decreased. The results represent the mean ± SEM of three different replicates and are representative of at least three different experiments, ∗*p* value < 0.05, ∗∗*p* value < 0.01, ∗∗∗*p* value < 0.001.

Following combination treatment for 48 h on MCF12F cells, the levels of *PUMA*, *p53*, and *Bax* mRNA were significantly decreased by ∼83%, ∼66%, and ∼44%, respectively compared to cisplatin treatment alone. In contrast, the mRNA levels of *Bcl-2* and *Bcl-xL* were increased ∼44.5% and ∼51%, respectively in the combination treatment compared to cisplatin alone. Also, the mRNA levels of BAX/Bcl-2 ratio was decreased by ∼81% in the combination compared to cisplatin treatment alone (Figure 5). Furthermore, combination treatment did not show any chemoprotective effect based on the mRNA expression levels of pro- and anti-apoptotic genes in MCF7 and MDA-MB-231 cells. (Figure 5). Amygdalin alone showed to increase p53 mRNA in MCF12F cells (Figure 5), however at the p53 protein level (Figure 4) remained unchanged and this might suggest that mRNA level does not always correlate to the protein level. Similarly, amygdalin did not promote cell death as assessed by flow cytometry in MCF12F cells (Figure 3).

## Discussion

The most widely used cancer therapies are surgery, radiotherapy, and chemotherapy, however, their effective therapeutic outcome remains limited due to their adverse side effects that can often be severe, thus highlighting the need for alternative or adjuvant therapies. Consequently, strategies that include phytochemicals may help in reducing these side effects and improve quality of life. Chemoprotection is a promising approach that aims at alleviating the chemotherapeutic side effects in the body (Maier et al., 2010). Phytochemicals seem to be involved in cancer prevention and treatment due to their relatively safe cytotoxicity profile (Duthie, 2007; Jagtap et al., 2009). In the last decade, progress studies aim to identify the potential of combinational strategies using one or more natural products along with an effective chemotherapeutic agent to enhance conventional cancer therapy (Krzyzanowska et al., 2010; Kaminski et al., 2011; Saldanha and Tollefsbol, 2012).

In this study, we investigated the chemoprotective and therapeutic action of amygdalin when combined with a conventional chemotherapeutic agent, cisplatin. To determine the differential cytotoxicity towards normal and cancer breast cells as well as in fibroblasts, the effect of varying concentrations of cisplatin and amygdalin, separately, was evaluated on MCF12F, MCF7, MDA-MB-231 cell lines, as well as on fibroblasts. Cisplatin and amygdalin decreased cell viability of all cell lines in a dose- and time-depended manner. It is important to note that amygdalin treatment of 10 mM in both normal and cancer cells did not show any statistically significant difference in cell viability and the rate was more than 90% in all time-points. This was supportive to previous findings indicating that many phytochemicals including amygdalin, Curcumin and Sulphoraphane are low-toxic for non-cancerous, normal cells (Ravindran et al., 2009; Sharma et al., 2011).

Based on the IC_50_ results the concentration of amygdalin was settled to be 10 mM (did not affect cell viability in any cell lines, in 24 h). The concentration of cisplatin was settled to be 15 μM for all cell lines in order to have more than 50% cell viability (to better evaluate the effect of combination treatment in normal cells).

Cisplatin is a well-known chemotherapeutic drug for the treatment of a broad range of human malignancies but it is associated with severe side effects, and non-specific cytotoxicity that is leading to normal cells damage and development of drug resistance (McWhinney et al., 2009; Gopal et al., 2012). Previous studies demonstrated the cytotoxic synergism of cisplatin and other agents such as bee venom, thiazolo[5,4-b] quinoline derivative D3CLP, and AT-101 drug towards various cancer cell lines (Alizadehnohi et al., 2012; Gonzalez-Sanchez et al., 2012; Mazumder et al., 2012; Karaca et al., 2013). Another study demonstrated the synergistic activity of phytochemical Epigallocatechin gallate (EGCG) in combination with cisplatin and tumor-active palladium compounds in ovarian cancer, suggesting that combinations of platinum drugs including cisplatin and designed trans-palladiums along with selected phytochemicals could mediate in overcoming drug resistance in the future. In our study we used a combination treatment of 15 μΜ cisplatin and 10 mM amygdalin both in normal MCF12F cells as well as FBS, to further evaluate the safety of amygdalin on normal cells as well as its cyto-protective abilities in the presence of anticancer treatments. The ability of amygdalin in sensitizing the death-inducing effects in cancer cells was also studied.

Flow cytometry confirmed the chemoprotective effect of amygdalin decreasing the % percentage of late apoptotic cells by up to 4.5-fold (Figure 3). As shown amygdalin was able to decrease the cytotoxic effect of cisplatin on normal breast cells. This is supported by the Combination Index (CI), which showed antagonism between amygdalin and cisplatin when used in normal cells and synergism against cancer cells.

Amygdalin gained wide popularity due to its anti-cancer activity and by other beneficial effects on different body systems such as inhibiting renal fibrosis, anti-asthmatic action and improving the immune function (Syrigos et al., 1998). However, the chemoprotective potential of amygdalin on normal-like breast epithelial cells has never been investigated before.

As our results indicated (Figure 5) the level of mRNAs of pro-apoptotic *PUMA, p53, BAX* were significantly decreased while the mRNAs of anti-apoptotic *Bcl-2* and *Bcl-xL* were increased following the combination treatment vs. cisplatin treatment alone in normal cells. This specific observation and finding supports the main rationale of the mechanism of action *via* the apoptotic pathway. In contrast, the level of mRNAs of pro-apoptotic *PUMA, p53, BAX* were increased while the mRNAs of anti-apoptotic *Bcl-2* and *Bcl-xL* were decreased following the combination treatment vs. cisplatin treatment alone in cancer cells. The results of RT-PCR in breast cancer cells (MCF7 an MDA-MB-231) suggest the possible synergistic effect of amygdalin with cisplatin and highlight its anti-cancer activity. It is known from previews reports that a possible mechanism for this toxic effect is the presence of *β*-Glucosidase in cancer cells but not in normal cells, that is able to cleave and release the cyanite from the main molecule of amygdalin. On the other hand, normal cells contain the enzyme rhodanese that cannot result to cyanide cleavage and release (Newmark et al., 1981).

A similar pattern of pro- and anti-apoptotic expression was also observed at the protein levels. The proteins expression of PUMA, p53, phospho-p53, and Bax (Figure 4) decreased as well, when a combination treatment was used vs. cisplatin alone. On the other hand, proapoptotic proteins Bcl-2 and Bcl-xL exhibited an increased tendency in the presence of amygdalin. Furthermore, when a combination treatment was used reduced levels of cleaved form of caspase 9 and PARP was resulted indicating inhibition of the apoptotic pathway. We are clarifying that even though some inconsistency can be observed as far as the GAPDH level outcome, considering the resulting outcome in all of the other proteins run for the experiment vs. GAPDH the inconsistency cannot be considered problematic. Furthermore, the difference of caspase-9-cleaved between lane 1 (control) vs. lane 3 (15 μM cisplatin) is very faint but this is a usual phenomenon in situations when there is an increased apoptotic result.

These results might indicate that the combination treatment was able to downregulate apoptosis vs. cisplatin treatment.

Considering the previous alterations both on mRNA and protein expressions, we can conclude that the combination treatment of cisplatin and amygdalin can possibly promote normal cell survival selectively, by inhibiting apoptosis only in normal cells. These observations and finding support our primary and main hypothesis and are compatible with other studies that indicated similar mechanism of action of amygdalin, in different cell lines or in animal models (Kwon et al., 2003; Chang et al., 2006; Chen et al., 2013; Makarevic et al., 2014; Su et al., 2014).

**FIGURE 6:**
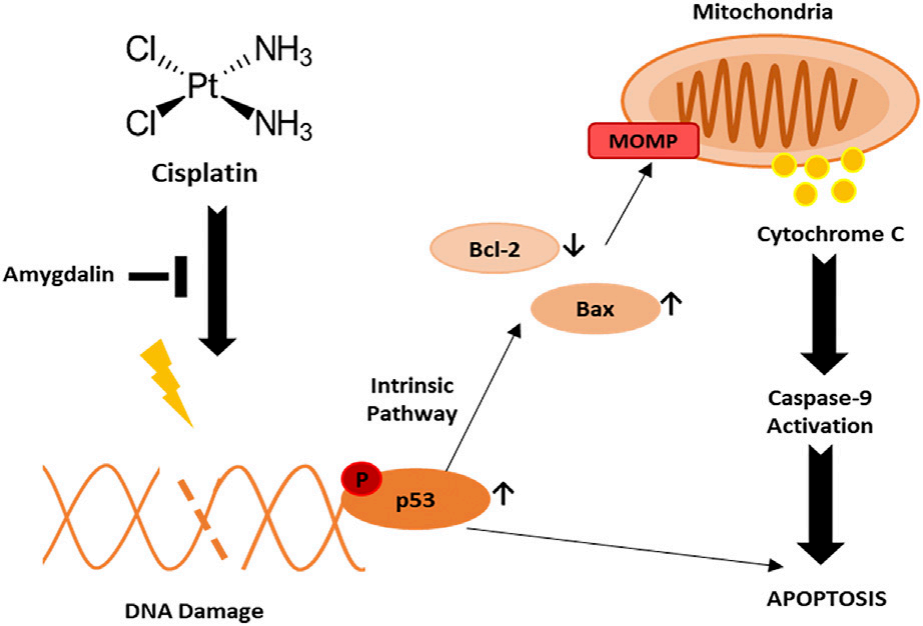
Potential mechanism of action of amygdalin and cisplatin in normal breast cell against breast cancer. Upon cisplatin treatment, DNA damage is induced followed by activation of phospho-p53 leading to apoptosis. Phospho-p53 may induce apoptosis through downregulation of Bcl-2 and upregulation of Bax leading to MOMP and activation of caspase-9.

In cancer cells, we showed that cisplatin promotes p53 activation leading to apoptosis through downregulation of Bcl-2 anti-apoptotic protein and upregulation of Bax pro-apoptotic protein. Pro- and anti-apoptotic Bcl-2 family proteins activate MOMP process which in turn activates apoptosis. MOMP results in the release of cytochrome c from the mitochondria into the cytosol, triggering caspase activation and subsequent apoptosis (Figure 6). On the other hand, amygdalin exerts a cyto-protective effect in non-cancerous cells by promoting an effective anti-apoptotic gene expression.

Breast cancer is known as one of the most lethal malignancies among women worldwide and it is major public health concern. Moreover, existing cancer chemotherapies are known to be associated with severe side effects affecting the quality of life of the patients and the life-expectancy as well. Thus, there is an urge need of new therapeutic approaches. This makes our present findings to have major importance contributing to the opening of new horizons and avenues in the field of cancer treatment.

This research could expand in the future by using *in vivo* experiments using animals to assess the efficacy of amygdalin in cancer treatment. Previous studies have shown that they treated mice with amygdalin using 200, 100, and 50 mg/kg body weight (Albogami et al., 2020). It is true that the concentration doses used (10–100 mM) can be considered very high for future animal studies. Still, the fact that they were not toxic gives us the confidence that they will not promote adverse or toxic effects when used in animals. Furthermore, we are clarifying that new dose optimization studies will be performed including PK studies for amygdalin detection in the plasma before use in animals.

Understanding the cytoprotective effects of amygdalin during chemotherapy may allow the development of novel therapies to reduce the adverse side effects and therefore improving the quality of life and life expectancy of cancer patients.

## Data Availability

The raw data supporting the conclusion of this article will be made available by the authors, without undue reservation.
