# Supplementary figures and images for: Amygdalin as a chemoprotective agent in co-treatment with cisplatin
Source: Front Pharmacol. 2022 Sep 20;13:1013692. doi: 10.3389/fphar.2022.1013692 (PMC9531591; doi:10.3389/fphar.2022.1013692)

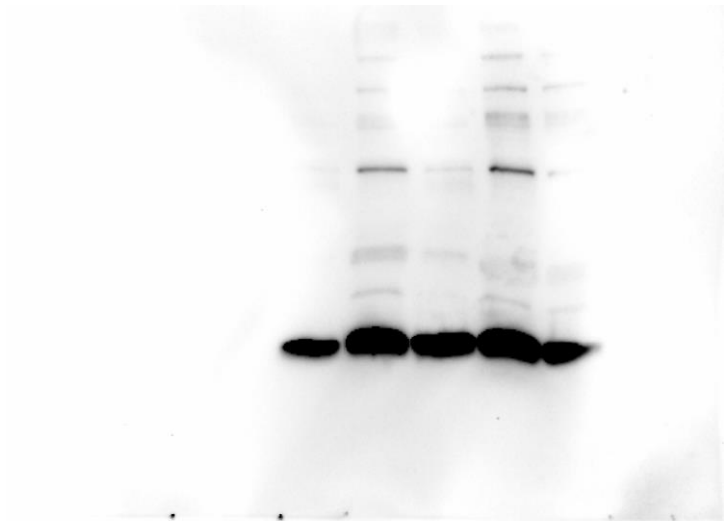

Bax

Bcl-2

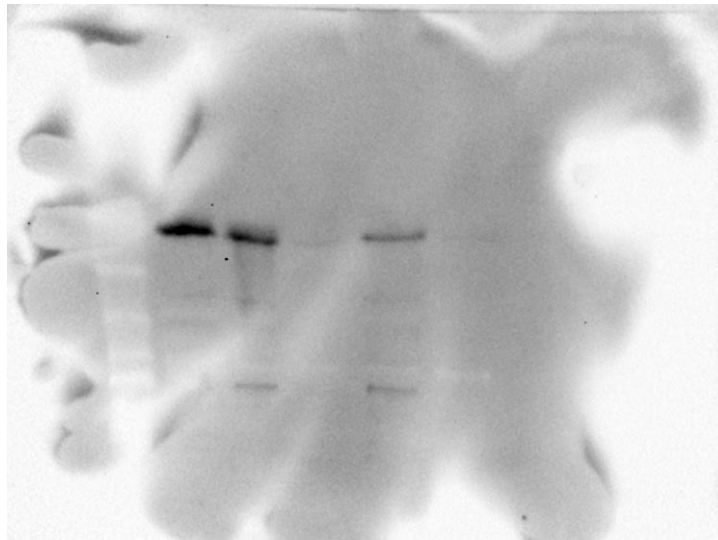

## Caspase 9

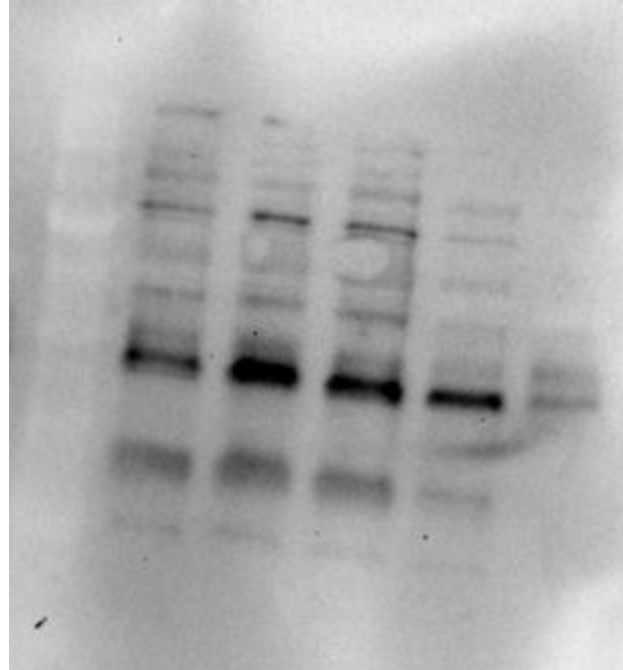

GAPDH

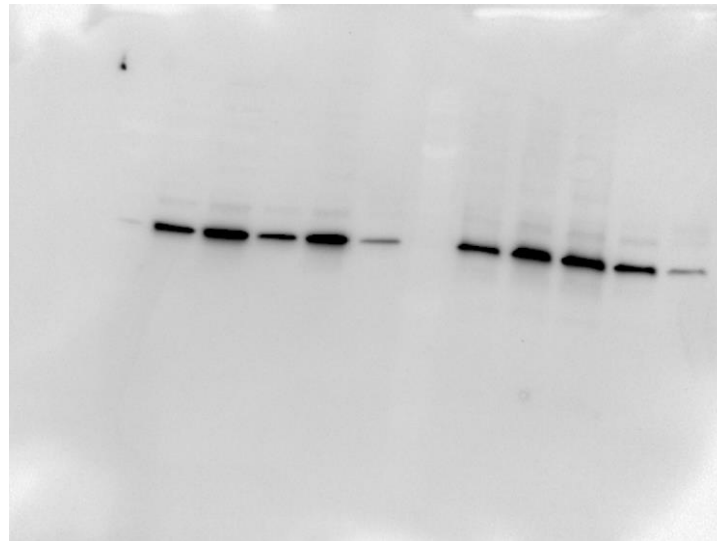

p53

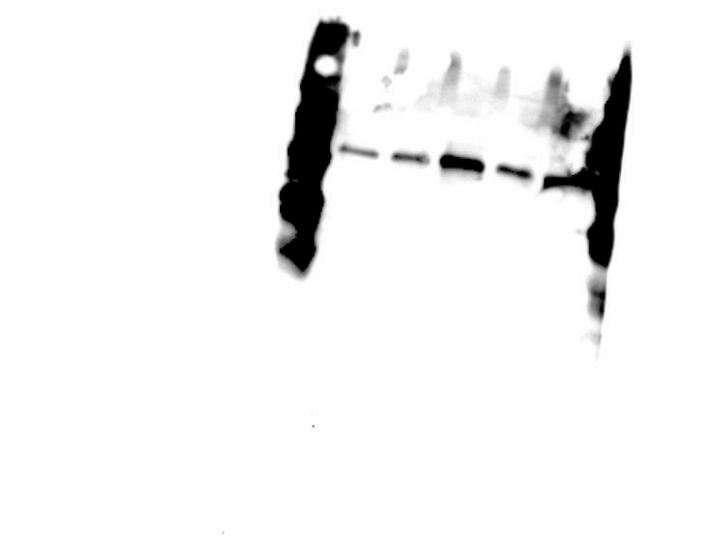

Phospho-p53

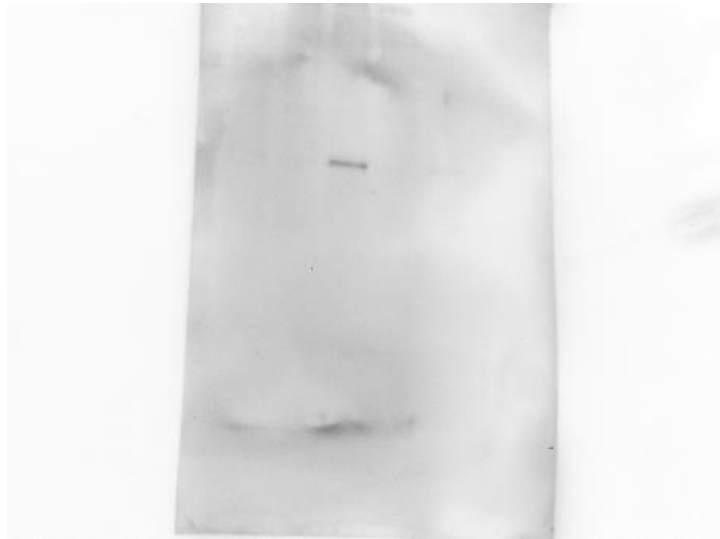

PUMA

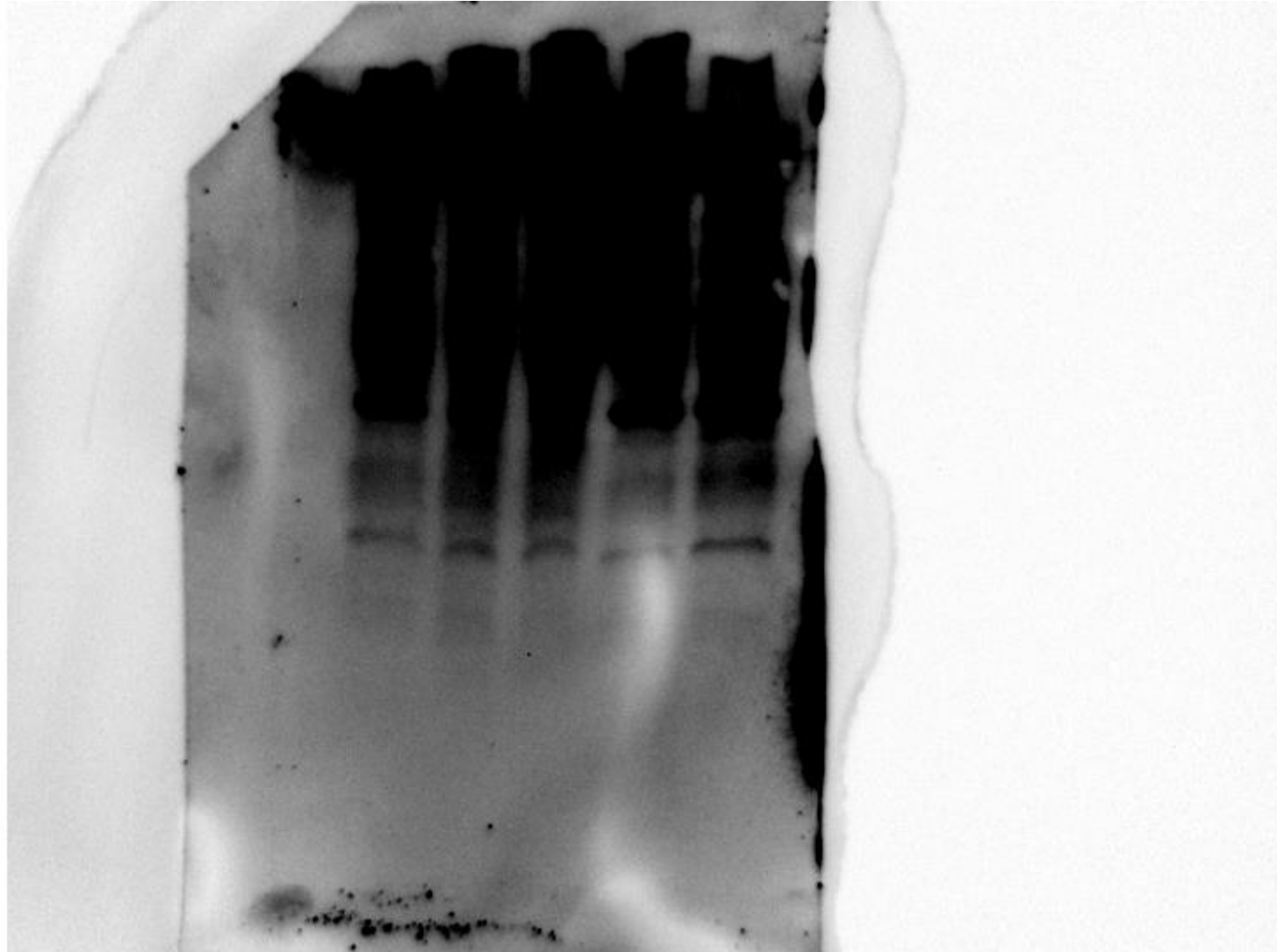

PURP

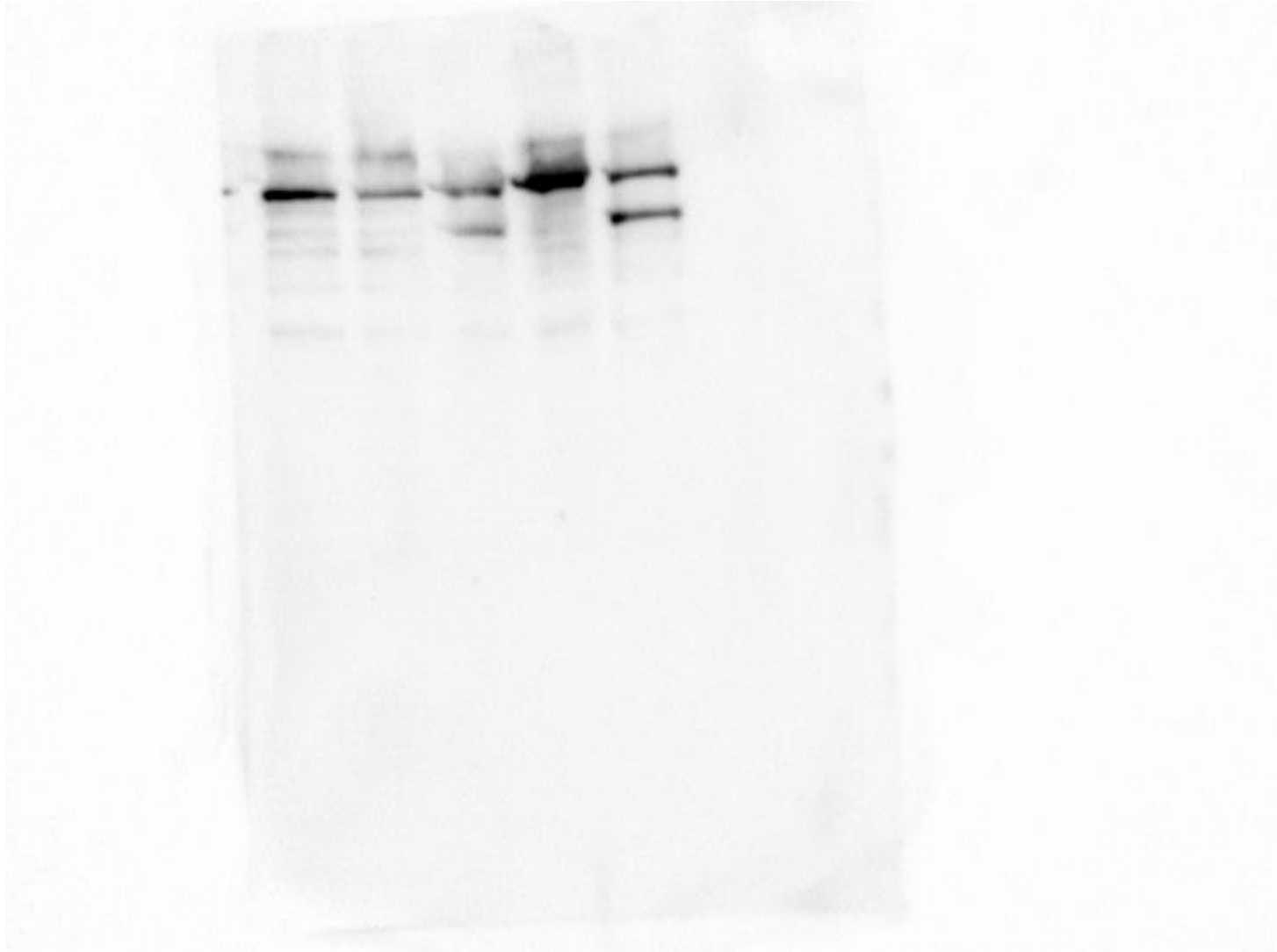

Supplement: Supplementary file 1 [file DataSheet1.PDF]

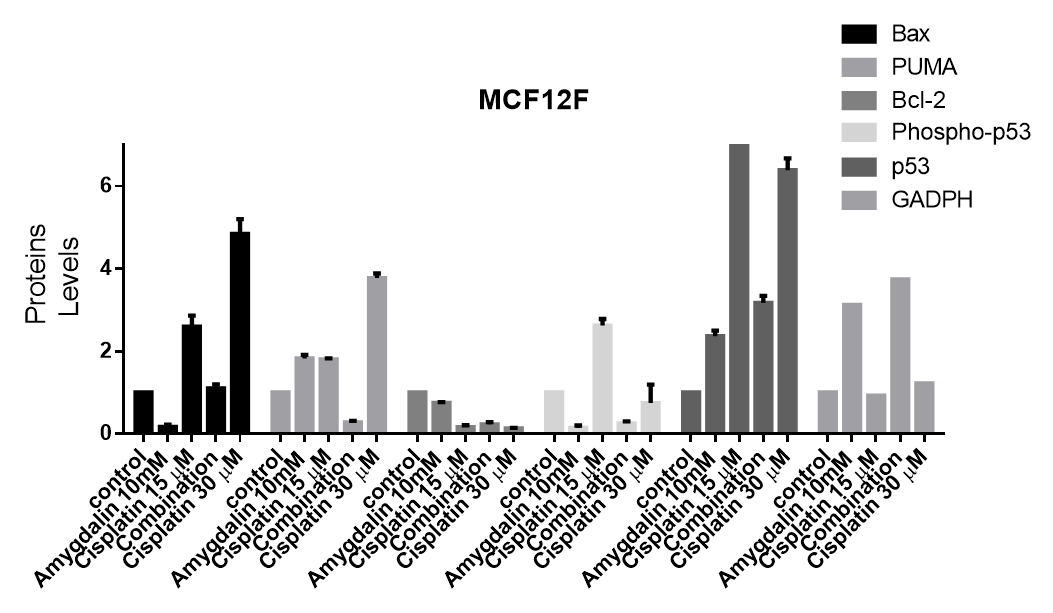

Supplement: Supplementary file 2 [file Image2.PNG]

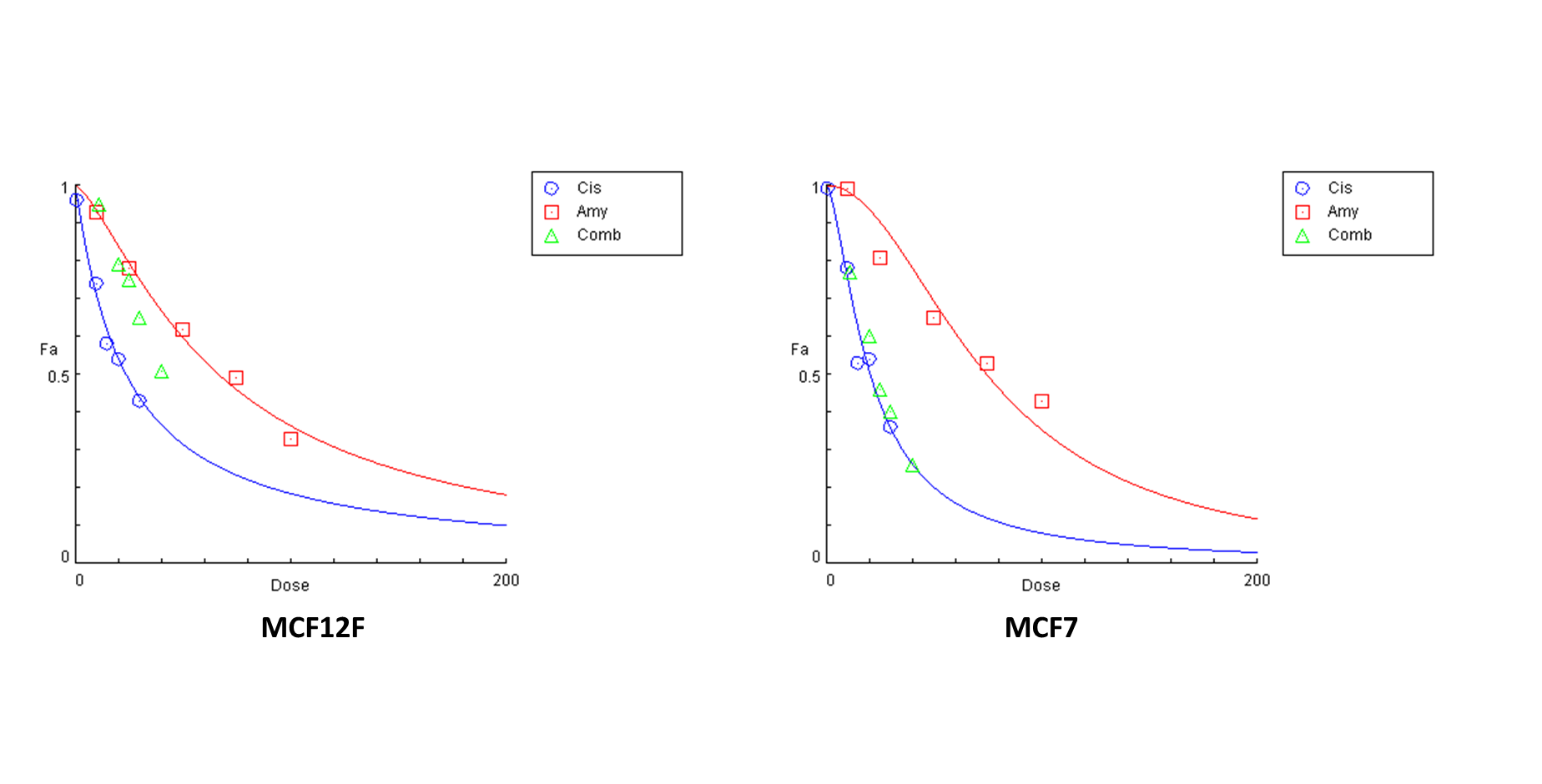

Supplement: Supplementary file 3 [file Image1.PNG]
